# What protects us against the COVID-19 threat? Cultural tightness matters

**DOI:** 10.1186/s12889-021-12161-1

**Published:** 2021-11-22

**Authors:** Dan Dong, Zhipeng Chen, Min Zong, Peng Zhang, Wen Gu, Yi Feng, Zhihong Qiao

**Affiliations:** 1 Faculty of Psychology, Beijing Normal University, No.19 Xinjiekouwai Street, Haidian District, Beijing, 100875 China; 2 Baotou School Affiliated To Beijing Normal University, Inner Mongolia, China; 3 Mental Health Center, China Foreign Affairs University, Beijing, China; 4 Mental Health Center, Central University of Finance and Economics, No.39 South College Road, Haidian District, Beijing, 100081 China

**Keywords:** Cultural tightness, Psychological disorders, Risk perception, COVID-19, Perceived protection efficacy

## Abstract

**Background:**

The only previous studies that formulated a theoretical model of epidemics for psychological response relative to cultural perspectives have focused on the role of individualism–collectivism and have omitted analysis of tightness–looseness. This study explored the role of cultural tightness in relation to psychological disorders during the outbreak of the COVID-19 pandemic.

**Methods:**

We recruited 1827 Chinese adolescents (*M*_*age*_ = 18.16 ± 2.23 years, 53.3% female) to participate a cross-sectional survey. Participants completed a series of questionnaires, including the scales of cultural tightness, risk perception of COVID-19 pandemic, perceived protection efficacy, anxiety and depression. A latent moderated structural equations model was used to analyse the mediating and moderating effects of risk perception regarding COVID-19, cultural tightness and perceived protection efficacy on psychological disorders.

**Results:**

The results showed that greater risk perception of COVID-19 predicted greater psychological disorders, however cultural tightness moderated this positive relationship. The increase in psychological disorders with risk perception regarding COVID-19 was less pronounced among people who lived in tighter cultural areas. In addition, this moderating effect of cultural tightness was further mediated by perceived protection efficacy; that is, tight culture protects against psychological disorders by enhancing perceived protection efficacy.

**Conclusion:**

This study enriched the theoretical framework of cultural tightness and indicated its importance in the field of mental health and health policies. It also emphasized the importance of tight culture as a protective factor against psychological disorders in case of COVID-19 outbreaks, providing valuable practical insight into psychological prevention for COVID-19 outbreaks.

**Supplementary Information:**

The online version contains supplementary material available at 10.1186/s12889-021-12161-1.

## Background

COVID-19, which was declared in January 2020 to be a public health emergency of international concern by WHO [1] has major impacts all over the world [2]. Fear of this overwhelming infectious disease has caused distress that is an unprecedented threat to psychological coping, leading to clinical and sub-clinical disorders, such as anxiety and depressive symptoms [2–4]. In fact, the incidence of psychological disorders such as depression and anxiety is not a sudden phenomenon. Before the outbreak of COVID-19 pandemic, the Chinese people are facing similar psychological disorders [5, 6] . The emergence of the COVID-19 may act as a catalyst to induce more people to be more susceptible to psychological disorders. Therefore, it is urgent to probe the factors that cause and relieve psychological disorders, and this effort would be crucial in protecting the public mental health in case of epidemic.

According to cognitive appraisal theory [7], the risk perception of COVID-19 is considered as a form of threat or risk event that leads to psychological disorders [8]. Risk perception is subjective and comprises multiple dimensions, mainly including appraised severity and controllability of risk [8]. People are aware of the severity of COVID-19 because of its lethality and uncontrollability due to its high infectivity [9]. However, not all people with high risk perception develop psychological disorders during a crisis event, because socio-cultural and psychological factors mutually affect psychological disorders [10]. Previous studies focused more on the social or psychological factors for mental health [11, 12], whereas a few studies suggested that cultural factors may serve as an antidote to mental health disorders in the pandemic. Furthermore, previous studies regarding the cultural factors mainly focused on the perspective of individualism-collectivism [10], while ignoring other dimensions of cultures, for instance, the cultural tightness-looseness.

As an important cultural factor, cultural tightness–looseness was first proposed by Pelto [13] as a set of unique cultural patterns that complement other measured cultural dimensions [13, 14]. It is defined as a shared structure and refers to the extent to which social norms are pervasive, clearly defined, and reliably imposed. Regions that have a culture with higher tightness tend to have historically suffered from famine, warfare, natural disaster, and disease, such as China and Japan [15]. Tight culture has two core characteristics: strong social norms and low tolerance for deviant behavior [14]. To fight a pandemic, governments in tight areas tend to formulate more stringent social norms and intervention policies to contain the spread of the disease, which may inhibit the spread of the COVID-19 [16], and alleviate potential psychological disorders. In addition, people in tight areas have greater self-monitoring, prevention self-guidance, and better self-regulatory strength, reflecting their adaptability to chronic situational restrictions [17], which may enable them less suffer from psychological disorders in the pandemic. It was proven that tight culture was effective in controlling the number of COVID-19 infections and deaths [16], but limited evidence regarding the effect of tight culture on mental health was found. Since cultural tightness generally links to a region’s public health policies in the pandemic [16], it is necessary to explore the role of cultural tightness on mental health, providing a reference on intervention strategies for other countries or regions.

Furthermore, it is necessary to study how tight culture, once established, relieves an individual’s psychological disorders in the pandemic. Tight culture may elevate individual perceived protection efficacy, which is the belief that individuals and groups can protect themselves from COVID-19 [10]. In general, individuals with higher perceived risk feel that they have less ability to cope with the virus [10]. However, tight interventions are effective in increasing the stability and controllability of the situation [16]. This can be expected to have improved people’s confidence in fighting the pandemic and led people to believe that the government has the ability to protect them from COVID-19, perhaps mitigating the reduction in perceived protection efficacy owing to the risk perception of COVID-19. In addition, protection–motivation theory proposes that the perceived threat of a health risk depends on psychological factors, including the ability to cope, and people with high perceived protection efficacy generate less psychological disorders [18]. Thus, we assume that the inhibitory effects of tight culture in the positive prediction of risk perception of COVID-19 on psychological disorders can be achieved by promoting perceived protection efficacy.

In summary, this study aims to build and examine a model that links cultural tightness and risk perception in COVID-19 with psychological disorders to explore the moderating effects of cultural tightness and its underlying mechanisms on psychological disorders. Based on these corollaries, we proposed the following hypotheses. First, greater risk perception of COVID-19 leads to greater psychological disorders, but cultural tightness moderates the relationship, such that the tighter the culture, this positive predictive effects would be attenuated. Second, perceived protection efficacy mediates the moderating effect of cultural tightness. In particular, cultural tightness can indirectly relieve people’s psychological disorders by weakening the negative predictive effects of risk perception of COVID-19 on perceived protection efficacy. Our research provided valuable practical insight into intervention strategies for public mental health from the cultural perspective in the pandemic.

## Methods

### Participants and procedures

This study adopted a cross-sectional design. It was conducted from March to April, 2020. We selected six classes from two majors in two universities in Anhui province; three classes from two majors in two universities in Jiangsu province; eight classes from two high schools in Liaoning province; and seven classes from two high schools in Inner Mongolia Autonomous Region. The minimum sample size needed is 10 times the number of estimated parameters in a structural equation model [19]. Thus, the minimum required sample is 560 in this study. A total of 2018 students participated in this survey, of which 191 students with missing data. Thus, the final sample size was 1827, with a response rate of 90.5%.

Students were invited to complete an online survey in the class by clicking a cellphone questionnaire link distributed by their head teachers who are in charge of their classes. Before participating in this survey, the head teachers were informed of the purpose and procedure of this study. Then the head teachers informed the participants about the purpose, procedure, and their rights to withdraw at any time, and they obtained informed consent from each participant. For students under the age of 18, their parents also provided informed consent. This research protocol was approved by the Research Ethics Review Committee of Beijing Normal University, China.

### Measures

#### Cultural tightness

The tightness–looseness scale was developed by Gelfand [15]. It was used in this study to measure the degree of individuals’ perception of the cultural tightness of the area in which they live using six items (see appendix). For example, “There are many social norms that people are supposed to abide by in the area where I live,” and “People almost always comply with social norms in the area where I live.” Item 4 was not appropriate in this study because it was negatively correlated with other five items (*r* = − 0.28 ~ − 0.54, *p* < .001) and the total score (*r* = − 0.34, *p* < .001), even if it was reverse coded; Thus, we deleted the item 4. Participant responses were given on a 6-point Likert scale, anchored at 1 (*strongly disagree*) and 6 (*strongly agree*). A composite score was calculated, with higher numbers indicating that people feel that the culture in the area where they live is tighter (Cronbach’s α = 0.78).

#### Risk perception of COVID-19

We used four items to assess individual risk perception for COVID-19, which were adapted from the Risk Perception of HIV Scale [20]. The adaptations made the items specifically relevant to COVID-19 rather than HIV. Two items assessed personal risk perception of COVID-19, namely, “I feel vulnerable to COVID-19 infection”, and “I worry about being infected with COVID-19”. Another two items assessed risk perception for the family, namely, “I feel that my family is vulnerable to COVID-19 infection”, “I worry about my family being infected with COVID-19”. All items were rated on 5-point scale, anchored at 1 (*never*) to 5 (*nearly every day*). We summed the scores to create a risk perception of COVID-19 composite (Cronbach’s α = 0.84), with higher scores reflecting higher risk perception for COVID-19.

#### Perceived protection efficacy

This study used three items to measure individual-perceived protection efficacy, following based on previous studies [10]. The adaptations made the items specifically relevant to COVID-19 rather than Ebola. One item was related to personal perceived protection efficacy (e.g., “I feel confident that I can protect myself from COVID-19”), one item was related to perception of the local region’s protection efficacy (e.g., “I feel confident that my local area can protect itself from COVID-19”), one item was related to perception of the country’s protection efficacy (e.g., “I feel confident that my country can protect itself from COVID-19”). Each item was rated on a 5-point Likert scale, ranging from 1 (*completely disagree*) to 5 (*completely agree*). The perceived protection efficacy composite was created by summing the scores (Cronbach’s α = 0.81), with higher scores indicating higher individual-perceived protection efficacy.

#### Psychological disorders

Anxiety and depression are commonly used as indicators for both the general population and in individual clinical practice to assess the level of psychological disorder [21, 22]. This study constructs psychological disorders as a latent variable composed of anxiety and depression. Anxiety symptoms were measured by applying the seven-item Generalized Anxiety Disorder Scale (GAD-7) [23]. The participants rated the frequency of anxiety symptoms over the previous 2 weeks on a 4-point Likert scale, with anchors at 0 (*not at all*) and 3 (*nearly every day*). The scores ranged between 0 and 21, with higher scores reflecting more serious anxiety symptoms. We set 5 as the cut-off score for screening for anxiety symptoms [24]. In this study, this scale was found to exhibit substantial reliability (α = 0.99). To measure depressive symptoms, we adopted the nine-item Patient Health Questionnaire (PHQ-9) [25]. The participants assessed their frequency of depressive symptoms over the previous 2 weeks on a 4-point Likert scale, with anchors at 0 (*not at all*) and 3 (*nearly every day*). Composite depression scores were created (Cronbach’s α = 0.92), with higher scores reflecting more serious depressive symptoms. We set 5 as the cut-off score for screening for depressive symptoms [25].

### Data analyses

The data analysis procedure was performed as follows: Initially, Spearman correlation analyses were conducted using SPSS 24.0. We utilized Mplus 7.4 to conduct the formal statistical analyses. The main variables were standardized before being used in the LMS model, and demographic variables were controlled as covariates. First, we built a latent moderated structural equations (LMS) model to test the moderating effects of cultural tightness on risk perception and psychological disorders. Second, to indicate the ways in which cultural tightness exerts the moderating effect, we set up a latent mediated-moderation structural model to determine whether perceived protection efficacy mediates the moderating effects of cultural tightness between risk perception and psychological disorders.

The overall fitness of the LMS model was assessed using a two-step method [26, 27]. First, we developed a structural model without the latent interaction, Model 0 (the null model, where latent interaction is not estimated). We utilized the *χ*^*2*^*/df*, the comparative fit index (CFI), Tucker–Lewis index (TLI), the root mean square error of approximation (RMSEA), and the standardized root mean residual (SRMR) to assess the fitness of this model. The acceptable criteria for the model were set as follows: CFI > 0.90, TLI > 0.90, RMSEA < 0.08, and SRMR < 0.08 [28]. Second, based on the good fit of Model 0, we built a structural model that included latent interaction, Model 1 (alternative model, latent interaction is estimated). The log-likelihood ratio test was used to compare the relative fit between Model 0 and Model 1 [29]. If the log-likelihood ratio test produces a significant value, this means that Model 0 represents a significant loss in fit relative to Model 1, meaning that Model 1 better fits the data [27]. We used the changes in R-squared (ΔR^2^) between Model 0 and Model 1 to detect the effect size of latent interaction [30].

## Results

### Demographic statistics and correlations

Of the 1827 participants (*M*_*age*_ = 18.16 ± 2.23 years), 46.7% were male (*M*_*age*_ = 18.06 ± 2.26 years). 45.3% were secondary school students, and 54.7% were college students. 49.3% of the participants were from city, 30.4% were from town, and 20.3% were from village; 16.8% were from Anhui province, 22.6% were from Jiangsu province, 23% were from Liaoning province, 20.9% were from Inner Mongolia Autonomous Region, 16.7% were from others province; 31.9% participants suffered from depression symptoms, 22.2% reported having anxiety symptoms. Table 1 displays demographic information of participants.

**Table 1:** Background characteristics of the participants (*N* = 1827)

	Without depressive symptoms (*N* = 1244)	With depressive symptoms (*N* = 583)	*p* value
	*n (%)*	*n (%)*	
Sex			0.260
Male (*N* = 853)	592 (47.6%)	261 (44.8%)	
Female (*N* = 974)	652 (52.4%)	322 (55.2%)	
School type			0.012
High school (*N* = 827)	588 (16.91%)	239 (41%)	
College (*N* = 1000)	656 (83.09%)	344 (59%)	
Province			0.002
Anhui (*N* = 307)	204 (16.4%)	103 (17.7%)	
Jiangsu (*N* = 412)	289 (23.2%)	123 (21.1%)	
Liaoning *(N* = 421)	300 (24.1%)	121 (20.8%)	
Inner Mongolia (*N* = 381)	271 (21.8%)	110 (18.9%)	
Others (*N* = 306)	180 (14.5%)	126 (21.6%)	
Location			0.481
City (*N* = 900)	624 (50.2%)	276 (47.3%)	
Town (*N* = 556)	375 (30.1%)	181 (31%)	
Village (*N* = 371)	245 (19.7%)	126 (21.6%)	
*Mean Age* (SD)	18.159 (1.944)	17.695 (1.962)	0.035
	Without anxiety symptoms (*N* = 1421)	With anxiety symptoms (*N* = 406)	*p* value
	*n (%)*	*n (%)*	
Sex			0.539
Male (*N* = 853)	658 (46.3%)	195 (48%)	
Female (*N* = 974)	763 (53.7%)	211 (52%)	
School type			0.074
High school (*N* = 827)	659 (46.4%)	168 (41.4%)	
College (*N* = 1000)	762 (53.6%)	238 (58.6%)	
Province			0.055
Anhui (*N* = 307)	228 (16%)	79 (19.5%)	
Jiangsu (*N* = 412)	326 (22.9%)	86 (21.2%)	
Liaoning (*N* = 421)	331 (23.3%)	90 (22.2%)	
Inner Mongolia (*N* = 381)	311 (21.9%)	70 (17.2%)	
Others (*N* = 306)	225 (15.8%)	81 (20%)	
Location			0.180
City (*N* = 900)	714 (50.2%)	186 (45.8%)	
Town (*N* = 556)	418 (29.4%)	138 (34.0%)	
Village (*N* = 371)	289 (20.3%)	82 (20.2%)	
*Mean Age* (*SD*)	18.12 (2.21)	18.30 (2.29)	0.164

*Note. p* value: Chi-square test and Mann-Whitney U test

The results of correlation analyses indicated that associations among psychological disorders (i.e., anxiety, depression) and risk perceptions of COVID-19 were positive (*r* = 0.27 ~ 0.28) and with cultural tightness and perceived protection efficacy were negative (*r* = − 0.05 ~ − 0.24). In addition, some demographic variables (i.e., age, sex, school type, dummy variable location 1, dummy variable province 2 and province 4) were significant correlated with psychological disorders (*r* = − 0.05 ~ − 0.24). Thus, we included above demographics in the model as covariates (*see* Table 2).

**Table 2:** Spearman’s correlations among the main variables

	1	2	3	4	5	6	7	8	9	10	11	12	13
1 Age	1												
2 Sex	0.04	1											
3 School type	0.89**	0.05*	1										
4 Location 1	0.02	0.04	0.05*	1									
5 Location 2	0.16**	−0.07**	0.18**	−0.33**	1								
6 Province 1	0.40	−0.02	0.47**	0.07**	0.12**	1							
7 Province 2	−0.57**	0.05*	−0.61**	0.05*	−0.04	−0.30**	1						
8 Province 3	−0.44**	−0.11**	−0.51**	−0.11**	−0.20**	−0.28**	−0.28**	1					
9 Province 4	0.33**	−0.04	0.33**	−0.04	0.16**	−0.24**	−0.25**	−0.23**	1				
10 Cultural tightness	−0.08**	−0.10**	−0.08**	−0.01	0.06*	−0.06**	0.12*	−0.02	−0.05	1			
11 Risk perception of COVID-19	0.01	0.07**	0.01	−0.02	0.01	−0.08**	−0.07**	0.06*	0.06*	−0.04	1		
12 Perceived protection efficacy	−0.01	−0.04	−0.02	0.03	−0.04	0.07**	0.06**	−0.08**	−0.08**	0.28**	−0.25**	1	
13 Anxiety	0.04	0.01	0.03	0.05*	0.02	−0.06*	−0.03	−0.02	−0.05*	−0.07**	0.27**	−0.20**	1
14 Depression	0.08*	0.05*	0.09**	0.04	0.02	0.00	−0.09*	−0.03	−0.09*	−0.11**	0.28**	−0.24**	0.73**

*Note.* Sex was coded as 1 = male, 2 = female. School type was coded as 1 = high school, 2 = college. Dummy variable location 1 was coded as 0 = city, 1 = town and location 2 was coded as 0 = city, 1 = village. Dummy variable province 1 was coded as 0 = Anhui, 1 = Jiangsu; province 2 was coded as 0 = Anhui, 1 = Liaoning; province 3 was coded as 0 = Anhui, 1 = Inner Mongolia; province 4 was coded as 0 = Anhui, 1 = others. *p < .05, **p < .01

### Latent moderating effect analyses

**Fig. 1:**
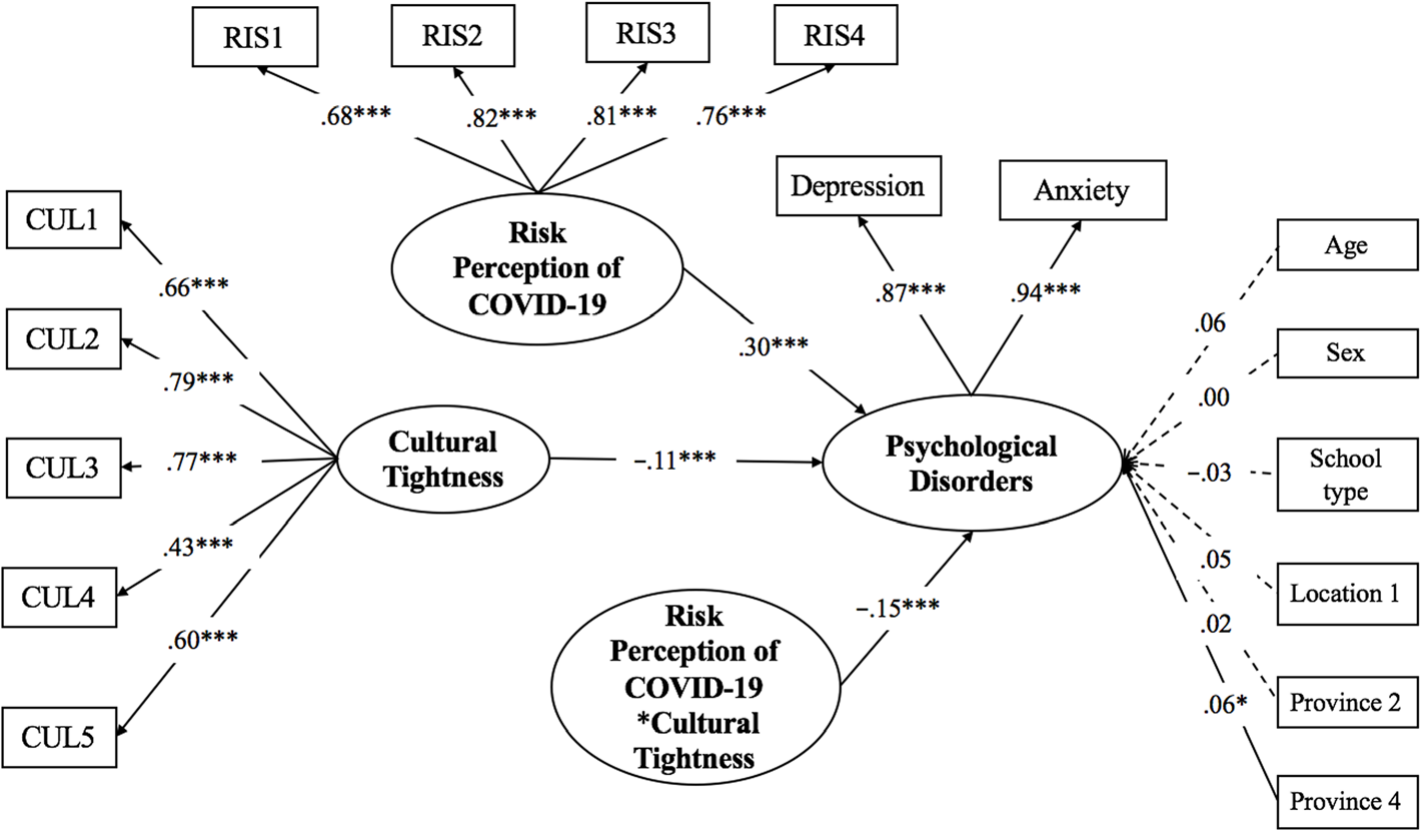
Latent moderated structural equations model for predicting psychological disorders. *Note.* CUL1-CUL5 are cultural tightness scale items; RIS1-RIS3 are risk perception of COVID-19 scale items. Age, sex, school type, location 1, province 2, and province 4 are controlled as covariates. *p < .05, ***p < .001

We investigated the moderating effects of cultural tightness on risk perception of COVID-19 and psychological disorders (*see* Fig. 1). First, we built Model 0 with latent variables cultural tightness and risk perception of COVID-19 as predictors, and the latent psychological disorders variable was set as the outcome. The demographic variables significantly correlated with the main variables were controlled as covariates. The results showed that Model 0 had good fit (*χ*^*2*^/*df* = 3.55, CFI = 0.97, TLI = 0.96, RMSEA = 0.04 (0.03–0.04), SRMR = 0.03). Second, using Model 0, we added the latent interaction of cultural tightness and risk perception of COVID-19 to build Model 1. In Model 1, the latent variable cultural tightness, the latent variable risk perception of COVID-19, and the interaction of cultural tightness and risk perception of COVID-19 were predictors, and the latent variable of psychological disorders was the outcome. Third, we run the log-likelihood ratio test and found that it was significant (D = 33.89, *df* = 1, *p* < .001), so we concluded that Model 1 with the latent interaction was well-fitted, and the effect sized of the latent interaction was 0.03.

**Fig. 2:**
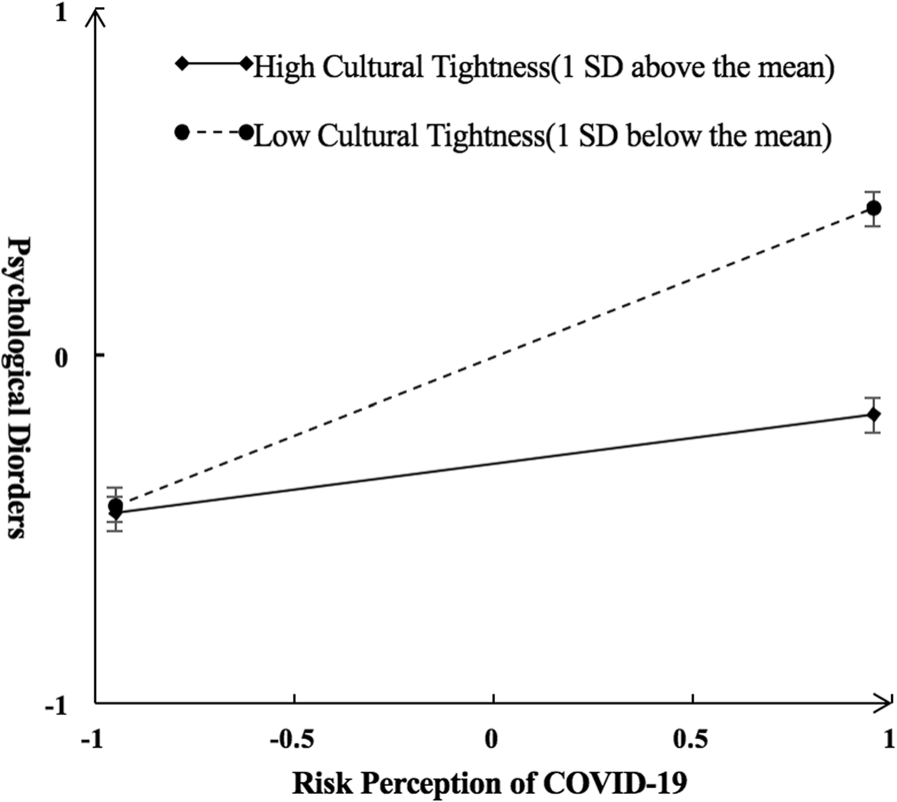
Interactive effect of risk perception of COVID-19 and cultural tightness on psychological disorders

In Model 1, greater risk perception of COVID-19 (*β* = 0.30, *p* = 0.001) and lower cultural tightness (*β* = − 0.11, *p* = 0.001) predicted greater psychological disorders. Moreover, the interaction effect between risk perception of COVID-19 and cultural tightness (*β* = − 0.15, *p* = 0.001) was a significant predictor for psychological disorders. Specifically, for low levels of cultural tightness (1 *SD* below the Mean), greater risk perception of COVID-19 predicted greater psychological disorders (*β* = 0.32, *p* = 0.001). However, at high levels of cultural tightness (1 *SD* above the Mean), the relationship between risk perception of COVID-19 and psychological disorders were less positive (*β* = 0.17, *p* = 0.001) (*see* Fig. 2).

### Latent mediated-moderating effect analyses

**Fig. 3:**
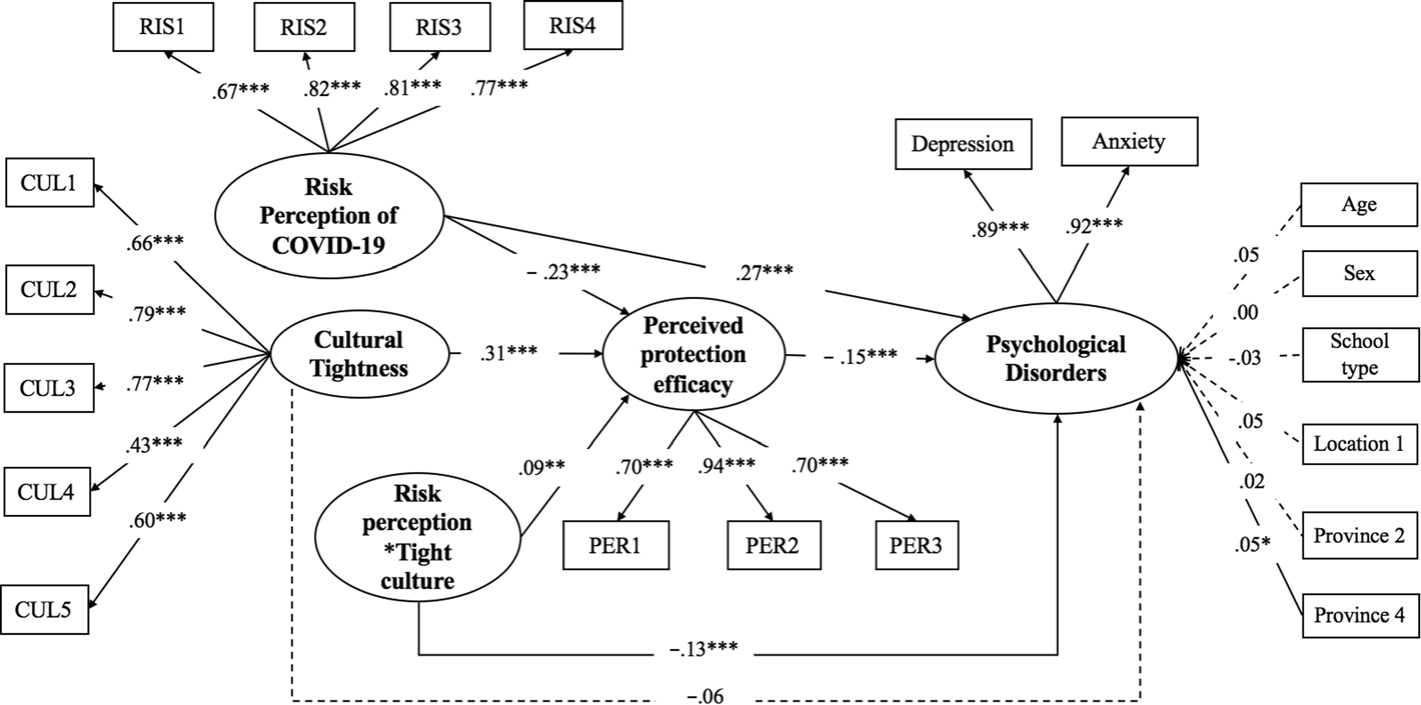
Latent mediated-moderation structural equations model for predicting psychological disorders. *Note.* CUL1-CUL5 are cultural tightness scale items; RIS1-RIS3 are risk perception of COVID-19 scale items; PER1-PER3 are perceived protection efficacy scale items. Age, sex, school type, location 1, province 2, and province 4 are controlled as covariates. *p < .05, **p < .01, ***p < .001

After evaluating the moderating effects of cultural tightness, we investigated whether the moderating effect of cultural tightness on risk perception of COVID-19 and psychological disorders was mediated by perceived protection efficacy. First, we ran Model 0 with the latent variable cultural tightness and the latent variable risk perception of COVID-19 as predictors, the latent variable perceived protection efficacy as mediator, and the latent variable psychological disorders as the outcome. Age, sex, school type, dummy variable location 1, dummy variable province 2 and province 4 were controlled as covariates. The results showed that Model 0 is a good fit (*χ*^*2*^/*df* = 3.25, CFI = 0.97, TLI = 0.96, RMSEA = 0.04 (0.03–0.04), SRMR = 0.03). Second, as the basis for Model 0, we added the interaction between cultural tightness and risk perception of COVID-19 as predictors to build Model 1 (*see* Fig. 3). Third, we conducted a log-likelihood ratio test, and the results showed that Model 1 with an interaction term fit the data better than Model 0 (D = 39.54, *df* = 1, *p* < .001), and the effect sized of the latent interaction 0.06.

**Fig. 4:**
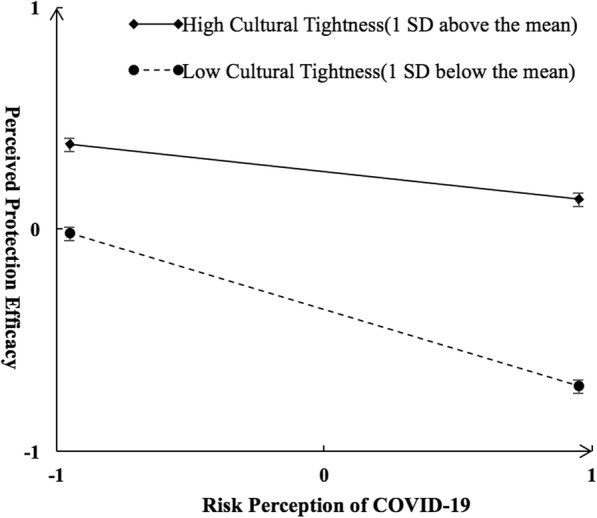
Interactive effect of risk perception of COVID-19 and cultural tightness on perceived protection efficacy

In Model 1, greater risk perception of COVID-19 (*β* = − 0.23, *p* = 0.001) and lower cultural tightness (*β* = 0.31, *p* = 0.001) predicted lower perceived protection efficacy, and the interactions between risk perception of COVID-19 and cultural tightness predicted perceived protection efficacy (*β* = 0.09, *p* = 0.002) (*see* Table 3). Specifically, at low levels of cultural tightness (1 *SD* below the Mean), greater risk perception of COVID-19 predicted lower perceived protection efficacy (*β* = − 0.28, *p* = 0.001). However, at high levels of cultural tightness (1 *SD* above the Mean), the relationship between risk perception of COVID-19 and perceived protection efficacy became less negative (*β* = − 0.14, *p* = 0.001) (*see* Fig. 4). Moreover, the lower perceptions of protection efficacy predicted greater psychological disorders (*β* = − 0.15, *p* = 0.001). Thus, the moderating effect of cultural tightness on risk perception of COVID-19 and psychological disorders was mediated by perceived protection efficacy. In addition, the interactions between risk perception of COVID-19 and cultural tightness also directly and significantly predicted psychological disorders after perceived protection efficacy was added (*β* = − 0.13, *p* = 0.001). Thus, the moderating effects of cultural tightness on risk perception of COVID-19 and psychological disorders were partially mediated by perceived protection efficacy.

**Table 3:** Fitness indices and standardized regression coefficients for the latent moderated structural equations model

	Latent moderated structural equations model		Latent mediated-moderation structural equations model	
	Model 0	Model 1	Model 0	Model 1
***Model fitness indices***				
*χ*^*2*^	358.31		484.38	
*df*	101		149	
Log (L)	−24,628.91	−24,611.96	−31,226.49	−31,206.72
CFI	0.97		0.97	
TLI	0.96		0.96	
RMSEA	0.04		0.04	
SRMR	0.03		0.03	
***Standardized regression coefficients***				
Risk perception of COVID-19 → psychological disorders	0.30***	0.30***	0.26***	0.27***
Cultural tightness → psychological disorders	−0.10**	−0.11***	−0.05	−0.06
Risk perception of COVID-19 × cultural tightness→ psychological disorders		−0.15***		−0.13***
Tight culture → perceived protection efficacy			0.31***	0.31***
Risk perception of COVID-19 × cultural tightness → perceived protection efficacy				0.09**
Perceived protection efficacy→ psychological disorders			−0.17***	−0.15***
Δ*R*^2^	0.03		0.06	

*Note.* **p < .01, *** *p <* .001

## Discussion

This study examined the effects of risk perception of COVID-19 on psychological disorders and found that increased psychological disorders was produced by higher risk perception of COVID-19. These results supported the findings of previous studies [31]. The negative impact caused by the amplification of risk perception of COVID-19 exceeded the direct impact of COVID-19 itself [32]. Therefore, even for people who do not suffer from COVID-19, the high risk perception caused by COVID-19 due to its high communicability also fueled psychological disorders.

Second, we explored the moderating role of cultural tightness between risk perception of COVID-19 and psychological disorders and found that cultural tightness moderates the positive predictive effect of risk perception of COVID-19 on psychological disorders. When people perceive their region to have a relatively tight culture, the increased psychological disorders triggered by risk perception of COVID-19 is reduced. Individuals in culturally tight areas who are chronically exposed to stronger situations have subjective experiences that indicate that their behavioral options are limited, their actions are subject to evaluation, and there are potential punishments that result from these evaluations [17]. Thus, self-regulatory strength is greater in culturally tight areas, which can prompt to inappropriate behavior to a certain extent [17], such as not believing rumors, reducing hoarding behavior, and paying less attention to negative news of the pandemic, leading to reduced psychological pressure or negative affect. Accordingly, psychological disorders could also be relieved.

Third, we investigated in depth the underlying mechanism of cultural tightness on psychological disorders. The hypothesized moderating role of cultural tightness was indirectly located through the mediating role of perceived protection efficacy. Specifically, the risk perception of COVID-19 was associated with decreased perceived protection efficacy, but this association was weaker among those who perceived that their region had relatively more cultural tightness. In addition, perceived protection efficacy significantly lessened individual psychological disorders. To our knowledge, tight areas put high value on COVID-19 response and formulated strict social isolation policies to combat it, such as forbidding gatherings and going out at will, requiring everyone to wear a mask wherever they go, deploying security checks and infrared thermometers, performing accurate positioning and tracking for people who need to leave their place of residence, and so on. In fact, within two months of the outbreak of COVID-19, outbreak response planning in some regions of China were able to effectively control the spread of the epidemic to a great degree [33, 34]. This greatly promoted perceived protection efficacy, which formed a buffer against the fear of COVID-19, and effectively prevented anxiety and depression.

Although this study found that, in China, tightening in the face of COVID-19 could alleviate psychological disorders, we do not encourage other countries to shift from looseness to tightness to alleviate psychological disorders in these circumstances. First, tight culture is a product of distal ecological and historical threat, including high population density, resource shortages, territorial conflicts, epidemics, and a harsh environment [16, 17]. We know that China is a culturally tight nation [35], therefore Chinese were able to quickly identify and accept the tight programming during the pandemic. However, countries that have experienced less ecological and historical threat tend to retain a loose culture, such as those of the United States and New Zealand [17]. Loose culture has existed in these countries for several hundred years, and people’s understanding, emotions, and behavior have developed under those circumstances. If a culture blindly shifts from looseness to tightness due to the pandemic, the psychological disorders created by the necessary cultural adaptation may be greater than that caused by the pandemic itself. Second, not all people in culturally tight countries will have reduced psychological disorders. We know that in addition to its tight culture, China also has a collectivist culture [36]. The tight culture of Chinese originated from this collectivism [37]. Thus, it may be the combined effects of tight and collectivist culture that produced psychological protection from the threat of COVID-19. Countries that are partial to individualism might not be able to use tightness as a buffer.

### Implications

Our study provided a certain degree of theoretical enlightenment. First, it linked socio-cultural aspects to public mental health, such as the potential impact of culture on mental health in relation to the natural opportunities of the epidemic situation. This is a new attempt to understand people’s mental health in relation to socio-cultural psychology, providing a new perspective for understand the influencing factors of psychological disorders. Second, this study considered mental health from the perspective of cultural tightness, an important cultural dimension that has received increasing attention from researchers recently. Our study deepened the understanding of the concept of cultural tightness and broadened its theoretical framework. Third, earlier studies have shown that collectivism can protect against epidemic threat, and our study broadened the investigation of the cultural role from another dimension of culture, namely, cultural tightness, which deepened our understanding of the various dimensions of cultural roles. Last, previous work, showed no consistent conclusions regarding the influence of cultural tightness on mental health. This study found that tight culture can inhibit the increase of psychological disorder caused by risk perception that is conducive to mental health during an epidemic. We clarified the effects of cultural tightness on mental health under specific circumstances.

Our study also provided practical guidance for the mitigation of psychological disorders under the conditions of an epidemic. On the one hand, this study found that it a high risk perception of the virus led to psychological disorders. We provided empirical evidence for this psychological intervention to buffer against psychological problems by lessening the perception of risk during the pandemic outbreak, such as reducing attention to negative news, making rational judgments about this news, maintaining a regular schedule, and cultivating hobbies to divert inattention. On the other hand, pandemics such as SARS and H1N1 have been occurring frequently in recent years, and we found that cultural tightness had an inhibitory effect on psychological disorders in a Chinese sample during a pandemic. The present study can be a practical reference for measures to take for future new public health threats, and it also provided a reference for psychological intervention and health polies in the context of Chinese culture. This study suggests that during a disease outbreak, tight measures such as self-isolation and social distancing, maybe an efficient effort to debate with COVID-19 in China.

### Limitations and future directions

Some limitations of this study should be noted. First, the object of the study only included the student group, and future research should be developed to add samples from other groups to verify the universality of our research. Second, this study found that, after the perceived protection efficacy was added, the moderating effects of cultural tightness on risk perception of COVID-19 and psychological disorders persisted. This suggests that the protective efficacy only partially mediated the moderating effects of cultural tightness, such that still other factors mediated the moderating effect, such as hope, time perspectives and sense of control. Future research should explore this potential mechanism to better improve the understanding the impact of cultural tightness on mental health. Third, cultural tightness is not only an individual-level cultural orientation but a regional-level dimension as well. Because most samples in this study were drawn from only four provinces in China, we analyzed cultural tightness only on an individual level. Future research should take into account all of the provinces of China and verify the model from a group perspective. Fourth, this study was carried in a Chinese context, and its results are only applicable to China. Therefore, caution is necessary when the findings are generalized to other cultural backgrounds. Future research should test the generalization of our models in other cultural contexts, especially in the western cultural context.

## Conclusions

This study explored the socio-cultural determinants of mental health in the pandemic by elucidating how cultural tightness affected psychological response and public mental health when facing the risk of COVID-19. It broadens the theoretical study of tight culture on mental health by expanding it to novel areas of socio-cultural psychology, and it also provides practical direction for psychological prevention during the COVID-19 pandemic. In the context of Chinese culture, a tighter cultural context and isolation policies may relieve public psychological disorders during the pandemic. It may be that a dose of protection efficacy can act as an antidote to public fear, anxiety, and depression in this type of situation.

## Supplementary Information

**Additional file 1: Supplementary materials.** Cultural tightness and pandemic

## Data Availability

The datasets used and analyzed during the current study are available from the corresponding author on reasonable request.
