# Supplementary material for: What protects us against the COVID-19 threat? Cultural tightness matters
Source: BMC Public Health. 2021 Nov 22;21:2139. doi: 10.1186/s12889-021-12161-1 (PMC8607057; doi:10.1186/s12889-021-12161-1)
Supplement: Supplementary file 1 — Additional file 1: Supplementary materials. Cultural tightness and pandemic [file 12889_2021_12161_MOESM1_ESM.docx]

**Supplementary materials：cultural tightness and pandemic**

**Cultural tightness–looseness scale**

The following statements refer to the area where you live as a whole. Please indicate how much the degree you agree or disagree with the following statements. Note that the statements sometimes refer to “social norms”, which are standards for behavior that are generally unwritten.

| Strongly Disagree | Moderately Disagree | Slightly Disagree | Slightly Agree | Moderately Agree | Strongly Agree |
| --- | --- | --- | --- | --- | --- |
| 1 | 2 | 3 | 4 | 5 | 6 |

1. There are many social norms that people are supposed to abide by in the area where I live.
2. There are very clear expectations for how people should act in most situations in the area where I live.
3. In the area where I live, people agree upon what behaviors are appropriate versus inappropriate in most situations.
4. People have a great deal of freedom in deciding how they want to behave in most situations in the area where I live. (Reverse coded)
5. If someone acts in an inappropriate way, others will strongly disapprove in the area where I live.
6. People almost always comply with social norms in the area where I live.

Scale origin: Gelfand MJ et al. (2011). Differences Between Tight and Loose Cultures: A 33-Nation Study. *Science, 332*(6033):1100-1104.

**Risk perception of COVID-19 scale**

The following statements refer to your feelings since COVID-19 happened. Please indicate how often you have the following statements according to your real feelings.

| Never | Sometimes | Half the time | Often | Nearly every day |
| --- | --- | --- | --- | --- |
| 1 | 2 | 3 | 4 | 5 |

1. I feel vulnerable to COVID-19 infection.
2. I worry about being infected with COVID-19.
3. I feel that my family is vulnerable to COVID-19 infection.
4. I worry about my family being infected with COVID-19.

**Perceived protection efficacy scale**

The following statements refer to the views on yourself, the local government, and the country since COVID-19 happened. Please indicate how much the degree you agree or disagree with the following statements.

| Completely Disagree | Disagree | Neither Agree Nor Disagree | | Agree | Completely Agree |
| --- | --- | --- | --- | --- | --- |
| 1 | 2 | 3 | 4 | | 5 |

1. I feel confident that I can protect myself from COVID-19.
2. I feel confident that my local area can protect itself from COVID-19.
3. I feel confident that my country can protect itself from COVID-19.
